# Whole-Genome Sequence of a Serotype 1/2b Listeria monocytogenes Strain Isolated from Raw Seafood in Japan

**DOI:** 10.1128/MRA.00206-19

**Published:** 2019-04-25

**Authors:** Chihiro Ohshima, Satomi Kanagawa, Satoko Miya, Ayaka Nakamura, Hajime Takahashi, Takashi Kuda, Bon Kimura

**Affiliations:** a Department of Food Science and Technology, Faculty of Marine Science, Tokyo University of Marine Science and Technology, Tokyo, Japan; University of Maryland School of Medicine

## Abstract

Listeria monocytogenes is a pathogen typically acquired through the ingestion of foods. It has been specifically reported that the pathogen is widely distributed in raw seafood in Japan. Here, we report the whole-genome sequence and sequence type (ST) of a Listeria monocytogenes strain isolated from salmon roe sold in the Japanese retail market.

## Abstract (precis)

Listeria monocytogenes is a pathogen typically acquired through the ingestion of foods. It has been specifically reported that the pathogen is widely distributed in raw seafood in Japan.

## ANNOUNCEMENT

Listeria monocytogenes is a Gram-positive facultative anaerobic rod-shaped bacterium known to cause the zoonotic infection listeriosis (1). The main symptoms are meningitis, sepsis, and abortion (2). Immunocompromised patients are particularly associated with severe listeriosis, and the fatality rate of this infection is 20 to 30%, which is high compared to those of other foodborne infections (3).

L. monocytogenes is cold and salt tolerant; therefore, it is widely distributed in certain environments, such as soils and rivers (1, 4, 5). Additionally, it has been isolated from many kinds of foods, e.g., cheese, milk, sausage, fruits, vegetables, and smoked salmon (6). It has been specifically reported that the pathogen is widely distributed in raw seafood in Japan (7, 8).

Here, we report the whole-genome sequence of L. monocytogenes strain 40-5-1, isolated from salmon roe sold in the Japanese retail market.

For strain isolation, 25 g of salmon roe, obtained from the retail market, was tested based on ISO 11290-1:2004, which is the method for detecting *L. monocytogenes* in foods as authenticated by the International Organization for Standardization (9). A typical colony on Palcam agar (Thermo Fisher Scientific, Inc., MA) was isolated and identified as L. monocytogenes by comparing the 16S rRNA gene sequence with the 16S rRNA sequence of L. monocytogenes in the blastn database. Additionally, the serotype of the strain was determined to be 1/2b by the agglutination method, using commercial Listeria antiserum (Denka Seiken, Tokyo, Japan) (7).

The genomic DNA of L. monocytogenes strain 40-5-1 cultured in Trypticase soy broth (Becton, Dickinson and Company, NJ, USA) was extracted by the phenol-chloroform extraction method (10). The genomic DNA was used to generate a sequencing library with an IonXpress Plus fragment library kit (Thermo Fisher Scientific, Inc.). Then, the sample was subjected to emulsion PCR, automatically loaded onto the Ion 318 Chip (Thermo Fisher Scientific, Inc.) by the Ion Chef system (Thermo Fisher Scientific, Inc.), and sequenced on the Ion PGM platform (Thermo Fisher Scientific, Inc.).

A total of 1,258,355 reads were obtained, and their average length was 294 bp. Reads were then assembled using SPAdes (version 5.8.0.0), with k-mer sizes of 21, 33, 55, 77, and 99, and the quality of the assembly was assessed using QUAST (version 2.3.). This resulted in 49 contiguous sequences longer than 100 bp, covering 3,028,715 bp in total, with a GC content of 37.83% and an *N*_50_ value of 238,762 bp. The maximum contig size and minimum contig size were 512,730 bp and 108 bp, respectively. The obtained data were submitted to the Multilocus Sequence Typing (MLST) database administered by the Pasteur Institute and the University of Oxford (11), and then the strain was identified as sequence type 5 (ST5) (clonal complex 155 [CC155]).

In the United States and Europe, where health surveillance systems are efficient, there are sufficient listeriosis data. Meanwhile, there were no surveillance data in Japan due to limited identification techniques and insufficient attention to L. monocytogenes in Asia (12). Hence, Asian data are not taken into account in global epidemiological surveys and comprehensive risk analyses. In the future, this report will play an important role in providing Asian data for large epidemiology surveys.

### Data availability.

The assembled genome sequence was registered in GenBank under the accession number BIXX01000000. Raw sequence reads have been deposited in the NCBI Sequence Read Archive under BioProject number PRJDB6714 and run number DRR124347.
